# The specific biochemistry of human axilla odour formation viewed in an evolutionary context

**DOI:** 10.1098/rstb.2019.0269

**Published:** 2020-04-20

**Authors:** Andreas Natsch, Roger Emter

**Affiliations:** Givaudan Schweiz AG, Kemptpark 50, CH-8310 Kemptthal, Switzerland

**Keywords:** human body odours, odour precursors, skin microbiome, axilla, aminoacylase

## Abstract

Human body odour is dominated by the scent of specific odourants emanating from specialized glands in the axillary region. These specific odourants are produced by an intricate interplay between biochemical pathways in the host and odour-releasing enzymes present in commensal microorganisms of the axillary microbiome. Key biochemical steps for the release of highly odouriferous carboxylic acids and sulfur compounds have been elucidated over the past 15 years. Based on the profound molecular understanding and specific analytical methods developed, evolutionary questions could be asked for the first time with small population studies: (i) a genetic basis for body odour could be shown with a twin study, (ii) no effect of genes in the human leukocyte antigen complex on the pattern of odourant carboxylic acid was found, and (iii) loss of odour precursor secretion by a mutation in the *ABCC11* gene could explain why a large fraction of the population in the Far East lack body odour formation. This review summarizes what is currently known at the molecular level on the biochemistry of the formation of key odourants in the human axilla. At the same time, we present for the first time the crystal structure of the *N*_α_-acyl-aminoacylase, a key human odour-releasing enzyme, thus describing at the molecular level how bacteria on the skin surface have adapted their enzyme to the specific substrates secreted by the human host.

This article is part of the Theo Murphy meeting issue ‘Olfactory communication in humans’.

## 1. Introduction

The human axillary skin region has long been recognized as an important scent organ. The axillary skin is covered by a dense array of glands [1], and at the same time, this skin niche is colonized by a large population of bacteria [2,3]. The skin glands secrete specific odourless precursor molecules, and the action of bacterial enzymes then triggers the release of the volatile odourants from these precursors. This complex interplay has been investigated at the molecular level in great detail over the past 15 years. Here, we summarize what is currently known on the biochemistry, focusing on both the glands of the host and the enzymes of the bacterial commensals. We present for the first time the crystal structure of the key enzyme involved in body odour release. We will then also review what is known on perception at the receiver side. The findings are finally integrated and discussed in relation to the role that axillary odour may have had in our evolutionary history and how coevolution shaped the enzymatic capacity of our commensal microorganisms on the skin.

## 2. Specific volatiles—human axilla odour is not a typical off-note

**Figure 1.**
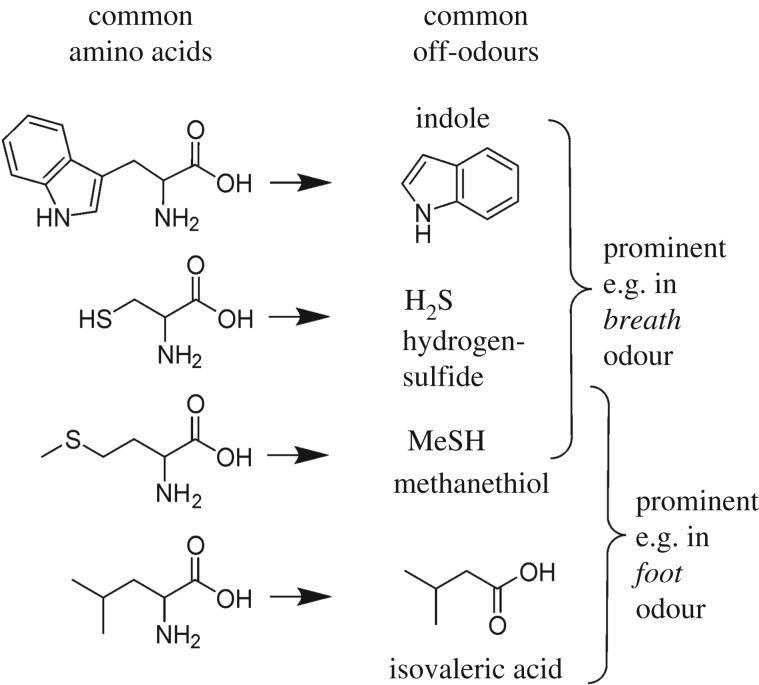
Components of typical offensive odours are often derived from simple bacterial catabolism of biological matter. Breath and foot odours are just two examples in which such amino acid degradation products play a part. The bacterial release of key axilla odourants is clearly distinct from these simple principles of malodour formation.

In today's Western society, axilla odours are perceived as unpleasant, and we commonly associate them with other unpleasant odours. Indeed, bacteria acting on organic matter are responsible for various offensive odours. Thus, anaerobic bacteria in the oral cavity cause bad breath or bacteria on the feet generate typical foot odours [4,5], while faecal odours are largely produced by gut bacteria. In all these instances, the volatiles are mainly generated from the bacterial degradation of common amino acids to volatile derivatives (figure 1) or from bacterial β-oxidation and abiotic oxidation of fatty acids leading to short-chain fatty acids, aldehydes and ketones.

The formation of axilla odour is completely different from these common catabolic processes: the odourant volatiles are very specific chemicals only occurring in the axilla, and the process of their release by bacteria is not part of the common bacterial primary catabolism.

**Figure 2.**
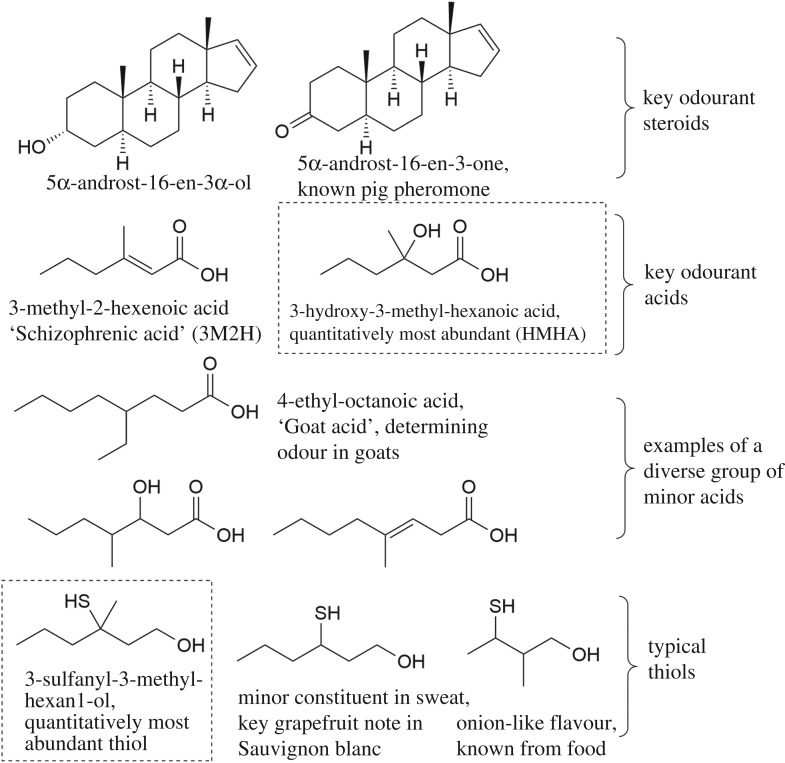
The known key human axilla odourants: note the common carbon skeleton of the most abundant acid and thiol highlighted with dotted boxes. These two volatiles had not been reported from other species or natural sources so far.

The most pungent odourants identified in axilla secretions are sulfur compounds, which were described by several research groups almost simultaneously [6–8]. 3-Sulfanyl-3-methyl-hexan1-ol is the most important odourant in this structural class. Quantitatively more abundant is a group of acids, with 3-methyl-2-hexenoic acid (3M2H) as a key [9] odouriferous principle identified first. Later, we could show that the closely related 3-hydroxy-3-methyl-hexanoic acid (HMHA) is quantitatively the most dominant human odourant [10]. These two carboxylic acids were so far not described from other species and hence might be human-specific. They are the most predominant examples of a more diverse class of branched or unbranched, saturated, unsaturated or hydroxylated odouriferous acids present in axilla secretions [11]. The key acids have carbon skeletons that are related to the sulfur compounds, suggesting a common biogenetic origin (figure 2). The third chemical group of odourants comprises two odouriferous steroids, originally described as key odourants and pheromones in the pig: 5α-androst-16-en-3-one (nicknamed androstenone) and 5α-androst-16-en-3α-ol (androstenol) [12,13]. However, these latter volatiles are present at very minor concentrations [14] and probably do not make a dominant contribution to the overall odour [15]. A third steroid, 4,16-androstadien-3-one (androstadienone), was also detected in one study [14]. Figure 2 depicts the structure of these key axilla odourants known today to contribute to the typical odour.

The specific nature and unique chemical structures of these odourants in figure 2 (versus the unspecific catabolism products common to other body odours and other malodours in figure 1) are a first indication that axilla odour is not a simple, random by-product of bacterial catabolism but may have had a function in chemical communication in an evolutionary context. The specific biosynthesis of such odourants and their targeted, localized secretion indicate that they did originally confer an adaptive advantage.

Both the acids and the sulfur compounds, which were investigated in most detail, had originally been identified based on their key contribution to the odour profile. This was achieved mainly using a gas chromatograph (GC) linked to a sniff-port, whereby a human panellist rates the intensity of individual components in the GC trace of a complex mixture [7,9–11]. Thus, their identification followed a classical ‘activity-guided fractionation’ principle rating their importance to the human receiver. Next to the chemicals identified by this method as key organoleptic contributors, a large number of other volatile chemicals are found in axilla secretions when investigated with comprehensive gas chromatography‐mass spectrometry (GC‐MS) analysis [16]. While these diverse volatiles may be of high interest as metabolomic markers, e.g. in forensics or in disease diagnostics, they are not necessarily important for the odour, as many may be odourless to the human nose. Other components may be odourant, but given their lower concentration or odour strength not make a key contribution to the overall odour. It is thus important not to equate volatiles and relevant odourants, as had been done in some studies [16]. To name an example, short, odourant, straight-chain fatty acids such as hexanoic acid, octanoic acid and isovaleric acid are clearly present in sweat, and their formation is not dependent on the biochemical pathways described below [17]. Thus, they were also identified in individuals in the Far East who did not having typical axilla odourants (see below) [17] or in prepubescent children [18]. A theory proposing incomplete β-oxidation of branched or uneven long-chain fatty acids as a crucial mechanism of axilla odour formation [19] was later revoked by the authors [15], who then agreed with the nature and biochemical origin of the key odourants as described in this review. Amino acid degradation products such as those presented in figure 1 will certainly be formed from dead keratinocytes, secreted proteins or free amino acids in the axilla by bacterial catabolism [18], but organoleptic assessment has not so far identified them as the key contributors to axilla odours of adult subjects with intact apocrine secretion while they are of key importance for bad breath and foot odour [4,5]. Both isovaleric acid and short, straight-chain acids may still be of importance in the weaker, acid odour noted in individuals lacking functional apocrine secretion (children and individuals lacking a functional *ABCC11* gene, see below) [18].

## 3. Odourant precursors

**Figure 3.**
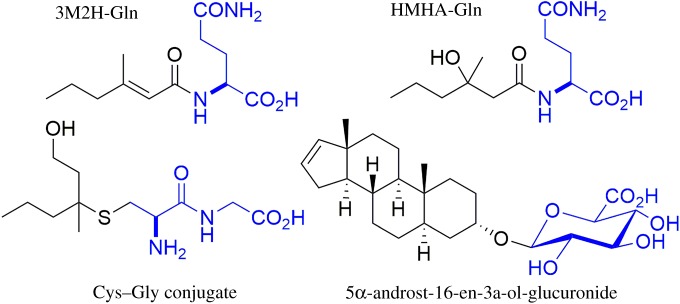
The structure of the three classes of odourant precursors: the volatile odourants are linked similarly to typical phase II metabolites produced in the liver: glutamine, Cys–Gly and glucuronide conjugates. (Online version in colour.)

All the odourants described above are secreted as odourless conjugates, and the structures of the precursors for all three classes could be determined (figure 3): all acids, both the two quantitatively dominant acids and also the complex array of minor acids, are linked to a glutamine (Gln) residue [10,11]. The sulfur compounds are secreted as part of a cysteine–glycine (Cys–Gly) dipeptide [20], and the steroid 5-α-androst-16-en-3α-ol was detected as a glucuronide conjugate in axilla secretions [21]. Such conjugates are well known from liver metabolism and toxicology: many acids are conjugated to Gln prior to secretion in the kidney, electrophilic chemicals are conjugated to glutathione and then secreted as Cys or Cys–Gly conjugates and many alcohols are linked to glucuronic acid to render them hydrophilic prior to secretion (e.g. [22,23]). While these toxicologically relevant conjugation reactions mainly occur in the liver, there is no indication that the odourant precursors discussed here are already formed by liver metabolism and then distributed systemically by blood circulation. With enzymatic treatment, we could not release the odourant acids from blood plasma, while they are readily released from axilla secretions [11]. This would indicate that these precursors are rather formed locally, and hence, the axillary gland is a specific metabolic organ synthesizing the odourant precursors for the local release in this scent organ—again pointing to an adaptive function of the axillary glands in human evolutionary history.

## 4. The importance of the skin microbiome

The anatomy of the axilla and the dense coverage by hair provide a moist environment; therefore, the population density and the diversity of skin bacteria are much higher in the axilla as compared to, e.g., the skin surface on the forearm [24]. A direct association between this bacterial population and body odour formation was early recognized: fresh apocrine secretions are odourless, but they develop the typical pungent odour if contacted with skin bacteria [25]. Two main bacterial genera colonize the axilla: *Staphylococci* and *Corynebacteria*, and odour formation was repeatedly associated with the population density of *Corynebacteria* [2,3].

The early work was performed with classical culture-based microbiological techniques. Over recent years, microbial populations have increasingly been analysed with culture-independent techniques based on high-throughput sequencing of the bacterial 16s-RNA gene. Such methods give a more detailed overview of the bacterial species present. In a correlation analysis between different genera present and odour strength and note, Troccaz *et al*. [26] identified key taxonomic groups relevant for odour release. These included the well-known *Corynebacteria*, but in addition they found that *Staphylococcus hominis* and anaerobic *Anaerococcus* species also correlate to odour formation*.* In principle, all those genera, whose abundance correlates to odour formation, would be expected to contain relevant enzymes for the cleavage of the odour precursors shown in figure 3. Next to the genera mentioned above, several studies on the axilla microbiome also reported members of the Propionibacteriae and *Micrococcus* sp. [26,27]. However, the abundance of these microorganisms did not positively correlate to odour strength, and thus, based on these correlative data, they appear not to be involved in the biochemistry of odour release.

The finally released and perceivable odour will always be determined by both the physiology of the host and the bacterial community. As will be described further below, a particular individual does secrete a specific and genetically determined ratio of the precursors. Therefore, different odours can, in principle, be formed in the presence of the same microbial population on different individuals. On the other hand, an individual may secrete precursors that are not or only partly cleaved by his or her commensal microorganisms, and in this case, the actual bacterial community composition has a profound impact on the finally released odour.

## 5. The biochemical steps for odour release—sulfur compounds

**Figure 4.**
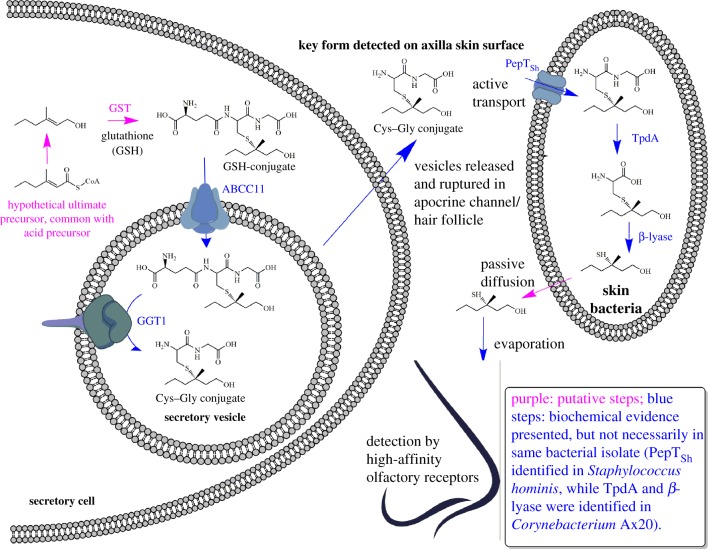
The key putative and established steps involved in the formation, secretion and bacterial release of sulfur compounds. GST, glutathione-*S*-transferase; ABCC11, multidrug efflux pump (ATP-binding cassette transporter sub-family C member 11); GGT1, γ-glutamyl transferase 1; PepT_SH_, peptide transporter *Staphylococcus hominis*; TpdA, thiol precursor dipeptidase A.

The release of the sulfur compounds has been studied in great detail by different research groups over recent years. Here, we present in figure 4 for the first time a comprehensive overview scheme of what is known and inferred by analogy on the biosynthesis and release of these volatiles.

At present, how the carbon skeleton of 3M2H is formed is unknown, but we may postulate a common precursor for both the key acids and the key sulfur compound: 3-methyl-2-hexenoyl-CoA. Hydrolysis and reduction of this precursor would generate 3-methyl-2-hexenol, which then would serve as a substrate for glutathione-*S*-transferase to form a glutathione (GSH) conjugate. This GSH conjugate has not been detected so far in biological samples from axillary glands, but common biochemical knowledge, especially from the toxicological field, indicates that a Cys–Gly conjugate—the finally detected form—is always a consequence of initial GSH conjugation [23].

Starting from this point, the sequence of events becomes less speculative, and the molecular details have been elucidated in great detail: the GSH conjugate is a substrate for the transport protein ABCC11 [17,28], which can transport the conjugate over the plasma membrane into the secretory vesicles of the secretory cells. Within the vesicle, the GSH conjugate becomes a substrate for the γ-glutamyl transferase 1 (GGT1), which removes the terminal glutamine residue, finally forming the Cys–Gly conjugate [28]. Release and rupture of the secretory vesicles brings this precursor into the secretory fluid and onto the skin surface.

Bacteria on the skin can take up the conjugate by active transport. This has been demonstrated for an isolate of *S. hominis*, and the uptake protein was characterized at the molecular level based on the crystal structure [29]. For *Corynebacteria*, the transport protein was not investigated, but we could show that the enzymes for the cleavage of the conjugate reside inside the cells [30] and hence active uptake is required, too. Indeed, it is only in *Corynebacteria* that the enzymes for the release of the sulfur compounds have been characterized so far. The Cys–Gly conjugate cannot directly release the volatile thiol. The sequential action of a dipeptidase removing the Gly residue followed by the release of the sulfur compound by a β-lyase is required [30]. We could clone and heterologously express the genes for both these enzymes to study the transformations at the molecular level. Once the low-molecular-weight thiol is released, it most likely passes through the bacterial cell membrane by passive diffusion finally to evaporate from the skin.

The complex sequence of events summarized in figure 4 illustrates how the formation of this odour is only possible through an intricate interplay between biosynthetic steps in the host and the selective release of the volatiles by the skin commensal bacteria that harbour highly specific enzymes for uptake and breakdown of the precursors.

## 6. The biochemical steps for odour release—acids

**Figure 5.**
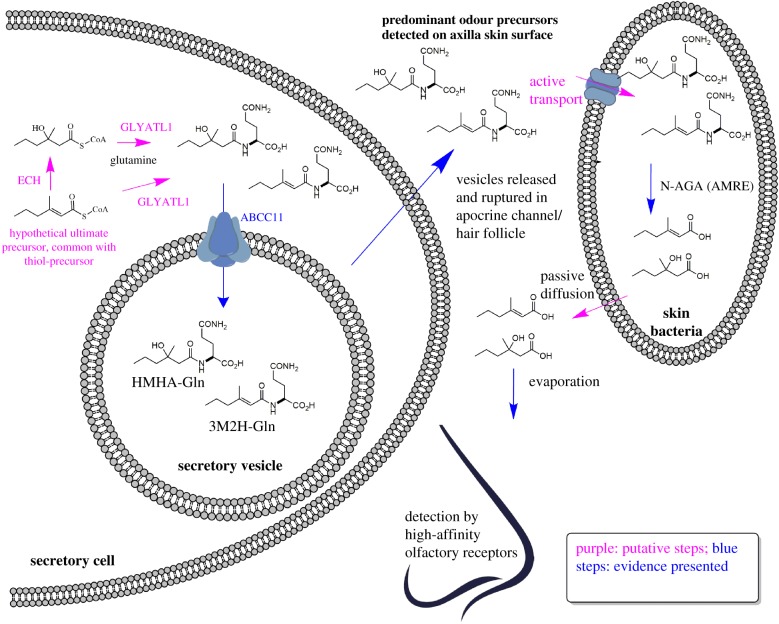
The key putative and established steps involved in the formation, secretion and bacterial release of volatile acids. GLYATL1, glycine-*N*-acyl-transferase like 1; ECH, enoyl-CoA hydratase; ABCC11, multidrug efflux pump (ATP-binding cassette transporter sub-family C member 11); N-AGA (AMRE), *N*_α_-acyl-glutamine aminoacylase.

The release of the acids is less complicated as compared to the sulfur compounds, but we can draw a similar overview scheme illustrating what is known and what can be inferred from common biochemical knowledge (figure 5). From the postulated common precursor 3-methyl-2-hexenoyl-CoA, *trans*-2-enoyl-coenzyme A hydratase, an enzyme involved in the degradation of short fatty acids by β-oxidation [31], could form 3-hydroxy-3-methyl-hexanoyl-CoA. Further steps in the β-oxidation sequence would then be blocked by the presence of the methyl group in β-position, blocking further catabolism of these CoA-esters. At the same time, these CoA-esters could serve as acyl donors for the enzyme GLYATL1 (Glycine-*N*-Acyl-transferase Like 1) [32], which is the only known human enzyme transferring acyl residues from CoA-esters to Gln. The Gln conjugates then appear to be substrates for the ABCC11 protein: while this was biochemically verified for the GSH conjugate with *in vitro* tests, the secretion of Gln conjugates by ABCC11 has not yet been shown *in vitro*. Yet, the fact that human subjects lacking a functional ABCC11 allele are unable to secrete these Gln conjugates strongly suggests that, indeed, they are a substrate for this transporter too (see below) [17]. Again, the Gln conjugates would then reside in the secretory vesicles and hence be secreted onto the skin surface by apocrine secretion.

Active uptake into the skin bacteria is required to reach the intracellular enzyme for conjugate cleavage, although the transport protein is unknown. Precursor cleavage is then performed by an *N*_α_-glutamine aminoacylase (N-AGA). We isolated this enzyme from a strain of *Corynebacterium striatum* that we had cultivated from axilla washings. In a small survey of isolates, we identified this enzymatic activity in strains of *Corynebacteria* only, but not in *Staphylococci* [10], in agreement with the correlation data associating odour formation with the population density of *Corynebacteria* [2,27]*.* N-AGA is the first enzyme involved in odour release in the axilla that had been isolated [10]. Hence, we also coined it with the trivial name AMRE [33], standing for the Axillary Malodour Releasing Enzyme. The gene for this enzyme was cloned and expressed in *Escherichia coli* too, allowing its detailed characterization. Interestingly, the same enzymatic activity was later also reported for an anaerobic strain of *Anaerococcus* sp. [34], a genus also found to correlate to odour strength by more recent taxonomic work [26] and that had escaped the aerobic cultivation techniques used in earlier studies.

## 7. *N*_α_-glutamine aminoacylase—the key enzyme for the release of acids

**Figure 6.**
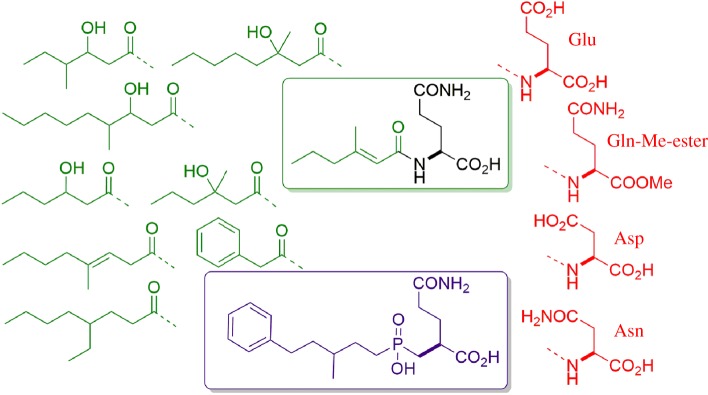
Substrate specificity of N-AGA (*N*_α_-acyl-glutamine aminoacylase). The enzyme is highly specific for the Gln residue, not accepting closely related amino acids (red), while specificity at the acyl part is loose, with all residues in green (natural substrates in the axilla) accepted. Based on this specificity, a high-affinity transition-state inhibitor (purple) was developed and used for co-crystallization with the enzyme.

The substrate specificity of N-AGA is telling an interesting story, as it points to coevolution of the enzyme in the commensal microorganism with the specific secretion of the human host: the substrate of the enzyme can be divided into the amino acid part and the acyl part. Interestingly, we found that only substrates with the Gln residue are cleaved, while analogues with closely related amino acids such as aspartate, asparagine or glutamate, or the methyl-ester of glutamate are all not recognized (figure 6). On the acyl side of the substrate, the enzyme is very flexible, and a wide number of linear and branched acyl chains, which may even contain a phenyl- or cyclohexyl-ring, are accepted. Thus, a highly selective transition-state inhibitor could be developed, that binds this enzyme with a *K*_i_ in the nanomolar range (figure 6). This inhibitor contains the substructure of Gln, the non-cleavable phosphinic group, which mimics the tetrahedral transition state of the amide group and a large acyl part for high-affinity binding [35].

Interestingly, this substrate selectivity nicely reflects the range of conjugates secreted in the axilla: we found a wide range of different odouriferous acids in complex axilla secretions (i.e. very diverse acyl parts) [11], but all are linked to the Gln residue (high specificity for the amino acid part). Thus, the substrate spectrum offered by the human host is reflected by the substrate specificity of the enzyme of the skin commensal. Knowing this unique substrate specificity, it became particularly interesting to investigate the interaction between the substrate and the enzyme at the molecular level. The availability of the N-AGA in pure form allowed a detailed characterization of this enzyme, and it was possible to resolve the crystal structure of the enzyme bound to the tetrahedral transition-state inhibitor at a resolution of 1.75 Å, which we report here for the first time.

### (a) Crystal structure of *N*_α_-glutamine aminoacylase

The gene coding for N-AGA [10] was expressed in *E. coli* and purified to greater than 99% purity by ammonium sulfate precipitation followed by passage over a phenylsepharose column and two passages over a Mono-Q column. A final concentration of 13 mg ml^−1^ of the pure enzyme was crystallized in presence of 10 mM of the competitive inhibitor (figure 6), 27% polyethylene glycol (PEG) 4000, 0.2 M sodium acetate, 0.09 M Tris (pH 8.5), 0.02 M ammonium sulfate, 0.01 M Bis–Tris (pH 6.5) and 2.5% PEG 3350. Crystals were grown by vapour diffusion in sitting drops at 23°C. X-ray diffraction was then measured at the X-ray source of the ESRF synchrotron (ID23-1 beamline).

The structure was resolved by molecular replacement using the two peptidases 1XMB [36] and 1YSJ as a search model. Based on this approach, the structure could be resolved at a resolution of 1.75 Å. Along with this publication, we deposit this co-crystallization structure in the protein bank PDB under accession number PDB ID 6SLF for detailed viewing.

**Figure 7.**
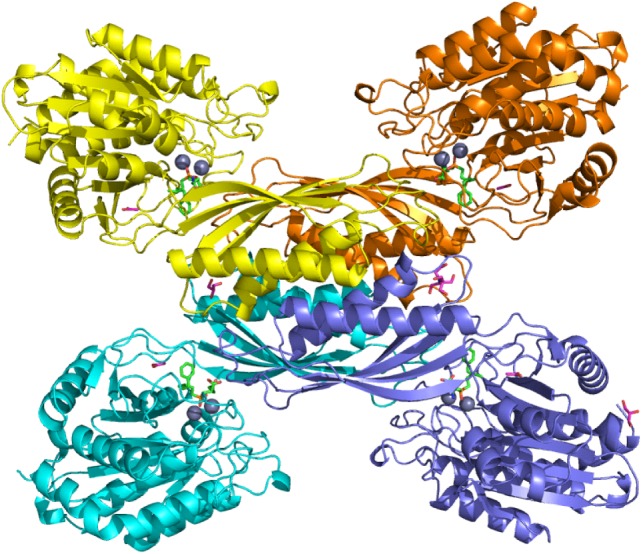
Overview of the crystal structure of N-AGA. Shown is the tetramer as it forms upon binding of the synthetic ligand and observed in the crystal structure. One dimer (yellow/orange) is a functional unit with each metal-binding domain binding two zinc atoms (blue balls) and one ligand (bright green).

Figure 7 shows the overall structure of the enzyme with its ligand bound. Similar to the related peptidases 1XMB and 1YSJ, the enzyme consists of a functional homodimer. It can be divided into a metal-binding domain and a dimerization domain, whereby a loop of the dimerization domain of the second monomer extends into the active site of the metal-binding domain, hence rendering the homodimer the functional catalytic unit. In the crystal structure, two dimers associate to form a tetramer (figure 7). We could show by size exclusion chromatography and native polyacrylamide electrophoresis that this is not a crystallization artefact, but that upon inhibitor binding, the enzyme forms a very stable tetramer also in solution [37] (although this may be an interaction specific to the chosen inhibitor class and not formed with the natural substrates).

**Figure 8.**
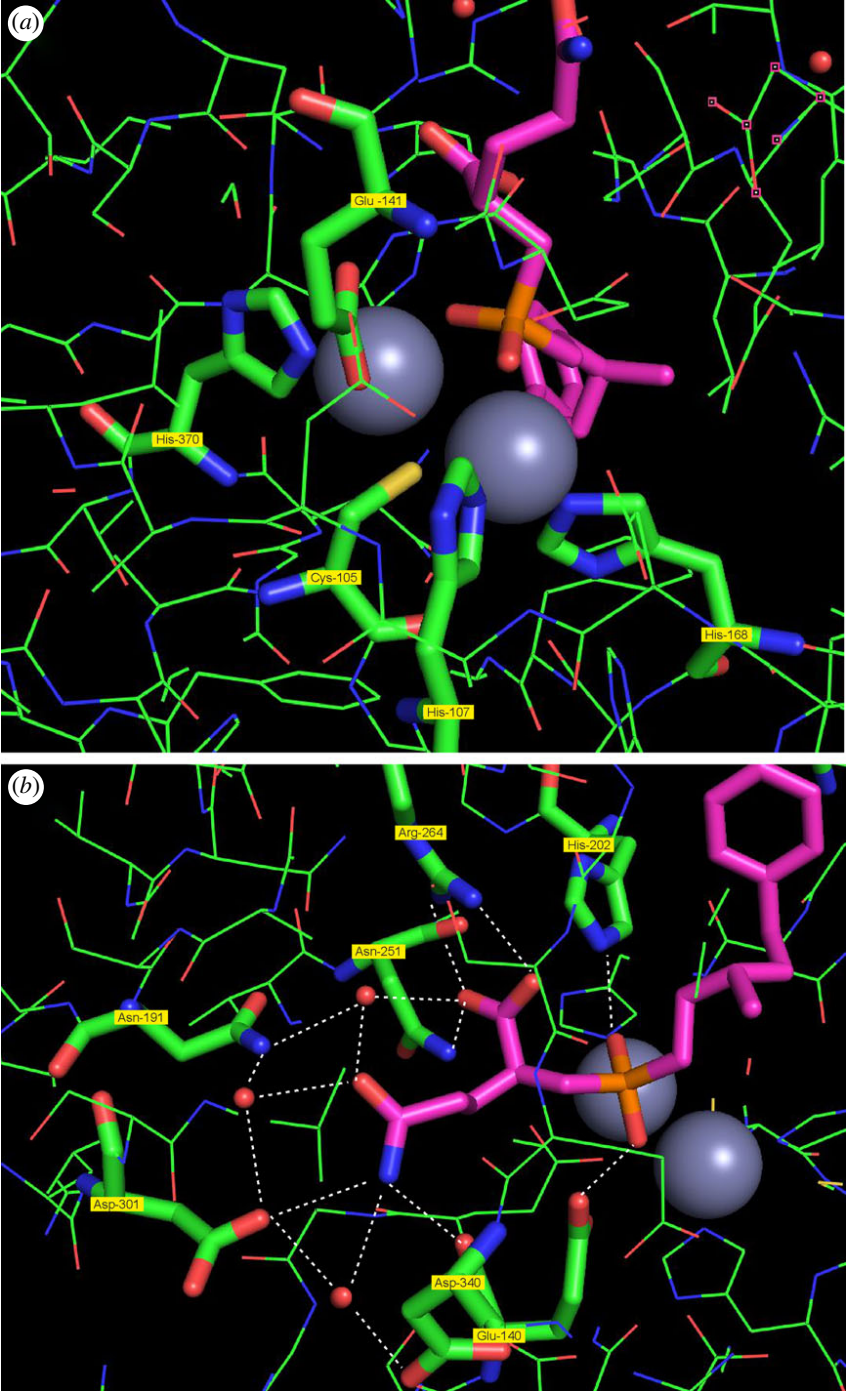
Close-up of the active site of N-AGA with the ligand bound. (*a*) The zinc-binding domain: the ligand (purple) is coordinated via the two oxygens of the phosphinic group (orange) with the two zinc atoms (grey). The zinc atoms are bound by the conserved Cys-105 coordinating to both zinc atoms and three conserved histidines (107, 168 and 370) and the carboxylate of Glu-141. (*b*) The domain binding the Gln side chain: multiple residues together with water molecules (red balls) form a dense network of hydrogen bonds (dotted white lines) specifically binding this side chain.

Each metal-binding domain contains two zinc atoms and one molecule of the inhibitor bound to the active site. Zooming in into the active site (figure 8), we can have a closer look at the catalytic centre (figure 8*a*). The two zinc atoms are tightly bound by three histidine residues (His-107, His-168 and His-370), and they are linked by a highly conserved Cys-reside (Cys-105) with the sulfur atom serving as a ligand for both zinc atoms. The carboxylate group of the conserved Glu-141 is a further coordination partner for one of the zinc atoms. The tetrahedral phosphinic group of the ligand is bound via its two oxygen atoms to the two zinc atoms offering to both of them a fifth coordination partner and mimicking the transition state of the substrate being cleaved.

Looking at the Gln side chain (figure 8*b*), we can observe a binding to several water molecules and to multiple residues of the apoprotein. A dense network of hydrogen bonds links the amide group of the side chain and the protein. Thus, the amide group forms hydrogen bonds to three water molecules that themselves are bound by hydrogen bonds to Asp-340, Asp-301, Asn-191 and the carboxylate group of the inhibitor. Furthermore, the amide group forms direct hydrogen bonds to Asp-301 and Glu-140. The free carboxylate group of the Gln side chain is bound by a salt bridge to Arg-264 and by a hydrogen bond to Asn-251. This part of the binding pocket is almost completely filled by the substrate. This tight binding of the Gln side chain by multiple bonds leaving no free space may explain the high adaptation of the enzyme to the Gln residues in the substrates (figure 6).

**Figure 9.**
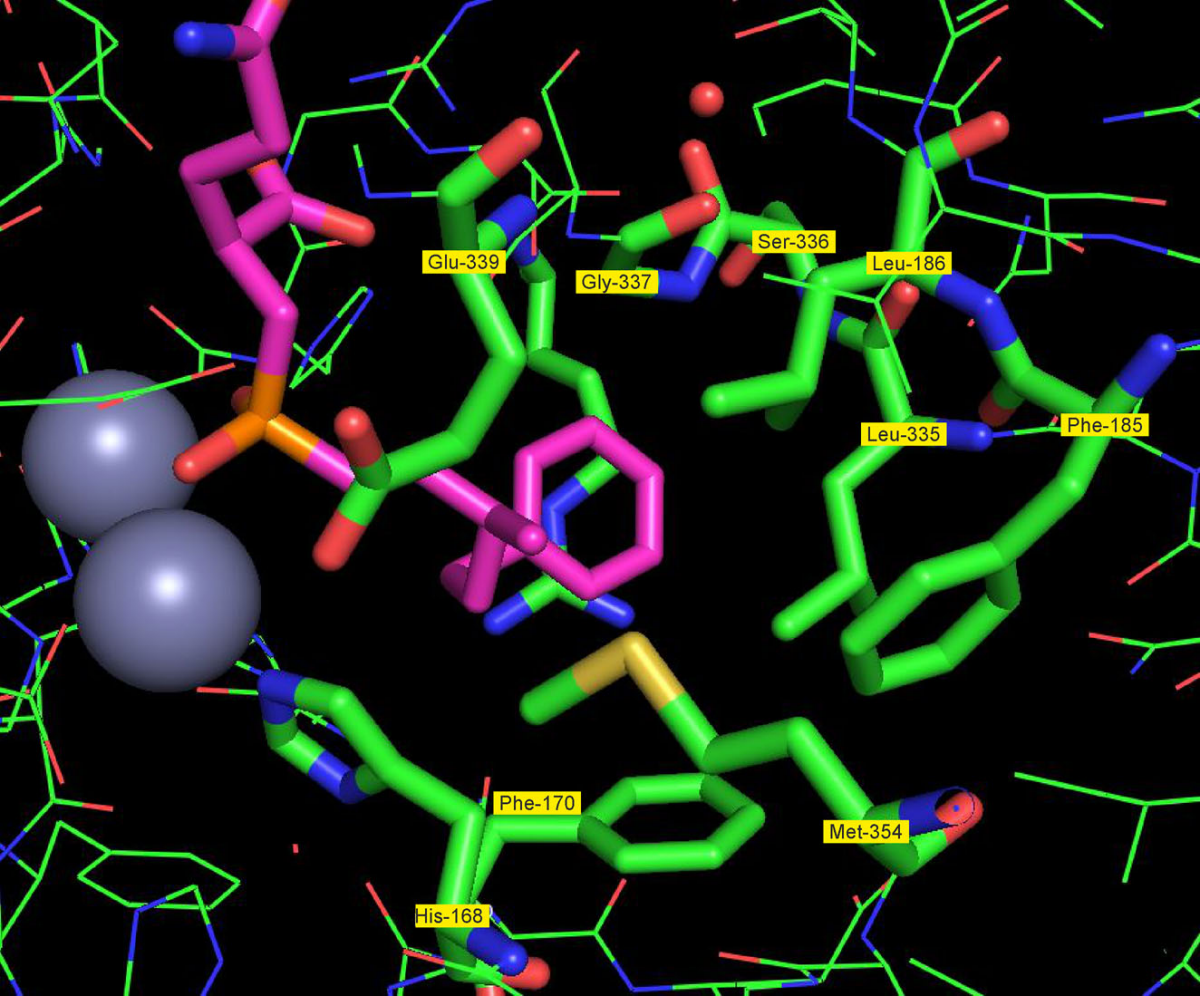
The hydrophobic pocket of N-AGA. Multiple hydrophobic residues (Phe‐170, Leu‐335, Phe‐185, Leu‐186, His‐168, Gly‐337 and Met‐354) line a large hydrophobic pocket binding the acyl part of the substrate and excluding any water molecules. Different acyl parts of the natural substrates and the larger acyl group of the synthetic inhibitor fit into this pocket.

On the other side, the hydrophobic part of the inhibitor resides in a large hydrophobic pocket (figure 9) formed by the side chains of Phe-170, Leu-335, Phe-185, Leu-186, His-168, Gly-337 and Met-354, excluding any water molecules. The hydrophobic part of the synthetic inhibitor is larger than the acyl part of most natural substrates, but still has ample space in this pocket explaining the low substrate specificity of the enzyme for the acyl residue, accommodating a wide range of natural and synthetic substrates.

## 8. ABCC11 mutations—an odourless genotype

As summarized above, odour precursor secretion into the secretory vesicles is catalysed by the efflux pump protein ABCC11. This finding is originally based on a unique observation in human population genetics: a significant fraction of the population in the Far East was long known to produce dry and white earwax, as opposed to the yellow and wet earwax dominant in the remaining global population. At the same time, individuals with the ‘dry and white’ phenotype were reported to lack typical axilla odour formation [38]. The ‘wet versus dry’ phenotypic difference is caused by a single-nucleotide polymorphism (SNP) 538G → A in the gene coding for ABCC11 [39]. By testing Caucasian and Asian volunteers for both this SNP and the secretion of the odourant precursors, we could indeed find a 100% association between at least one functional allele of the *ABCC11* gene and the presence of the amino acid conjugates in axilla secretions [17], suggesting that ABCC11 is directly involved in the secretion of the odour precursors from the apocrine glands. These results were later replicated in an independent study [38], and as indicated in figure 4, the GSH conjugate of the sulfur volatile was shown indeed to serve as a substrate for ABCC11 by biochemical tests [28].

The ABCC11 538G → A SNP is unique inasmuch as it is present on an extended haplotype, suggesting that the mutation occurred once and then spread rapidly in the population of the Far East in the form of this specific haplotype that has reached almost 100% prevalence in certain populations [39]. Therefore, a strong positive selection pressure for the phenotype resulting from the *ABCC11* mutation has to be inferred. The mutation does affect the composition of apocrine secretions in the ears, the axilla and also of the colostrum in early lactation [40]. It is therefore not obvious which loss of apocrine secretion in the mutated phenotype actually led to this adaptive advantage and the strong positive selection. Yet, it is tempting to speculate that an odourless phenotype became a preferred social attribute early on in ancient Asian cultures and hence did confer an advantage in mate selection. This would be an elegant (or simplistic?) explanation for the rapid spread of this haplotype in a large population. Furthermore, the high prevalence of the mutated ABCC11 (and therefore a lack of axilla odours) clearly highlights that axilla odours had lost their potential adaptive advantage in social interaction in those cultures where the mutant allele became prevalent.

## 9. An individual-specific odourtype

As indicated above, the N-AGA enzyme releasing odouriferous acids has broad substrate specificity for the acyl part of the substrates, and a wide variety of Gln conjugates are secreted by the host and cleaved by this bacterial enzyme [11] (figure 6). We were interested whether the composition of the specific mixture of released odour precursors may reveal any information on an individual based on a genetic determination of the odourtype or whether the specific cocktail of odourants released is rather a reflection of lifestyle factors such as diet, which may provide for different fatty acid precursors. To answer this question, axilla secretions of monozygotic twins were sampled, treated with N-AGA and then analysed by comprehensive 2D-GC–MS quantifying the pattern of 26 different acids released by N-AGA. This experimental approach (i.e. treating the secretions with an excess of the recombinant enzyme) does exclude any influence of the skin microbiome and allowed investigation of the effect of host genetics on the profile of odourants, thus excluding lifestyle and immunological factors that may affect the bacterial population. The patterns observed were stable when the same individual was tested twice and highly similar in the pairs of twins, suggesting that, indeed, this pattern is individual-specific and genetically determined [41]. Interestingly, all individuals have the two dominant acids HMHA and 3M2H (figure 2) as the main odourants, and the subtle difference between individuals is determined by the minor components. While we do not know to which extent humans in prehistoric societies used odour as a contributing cue in individual recognition, this genetically determined pattern of odour release at least indicates that there would be a biochemical basis for such behaviours. The dominant odourants present in all individuals may have composed ‘the human scent’ marking the presence of any conspecific, while the genetically determined minor components may have served in a more subtle way for communicating about individuality.

## 10. The quest for an odourtype linked to HLA genes

The question about a potential individual-specific odourtype has always been linked to the hypothesis that such an odourtype might be linked to genes in the HLA locus (coined MHC in other vertebrates) [42,43]. This locus codes for cell surface proteins required for the immunological defence against pathogens, and it is a key factor in transplant rejection [44]. The hypothesis for a link between body odours and these immune proteins is plausible based on the observation that inbred mouse strains differing only in the MHC locus can recognize and preferably mate with inbred mouse strains with different MHC alleles [45]. If indeed such a link between odour, odour preference and MHC did exist, an odourtype would not only inform about individuality but also guide mate selection to avoid inbreeding and to sire offspring with diverse MHC genes, thus having an advantage in pathogen defence. This potentially important contribution of an individual and MHC/HLA-dependent odourtype in evolution and mate selection has triggered high interest also regarding human scents. In a seminal study [46], the odour of T-shirts worn by men was smelled by several women, and the HLA type of both the T-shirt wearers and the sniffing individuals was analysed. Women were asked whether they prefer either smell of MHC-dissimilar or MHC-similar men and a preference for the smell of MHC-dissimilar men was found, but only in women not using hormonal contraceptives.

This led us to investigate whether the genetically determined odourtypes indeed would be determined by identical genes at the HLA locus [47]. We cannot summarize the experimental details of that study here—yet the key conclusion was that HLA-identical human siblings do not have highly similar patterns of released odourants and do not differ significantly from pairs of siblings not sharing any HLA haplotype [47]. This resembles the situation in mice, where despite a decade-long quest for odourant patterns linked to HLA alleles, no chemical correlate to the observed behavioural effects was found [48].

In a recent critique [49], we contributed some questions to this field: in mice, the behavioural evidence comes mainly from the comparison of inbred strains—however, the ability to olfactively discriminate inbred strains differing at a given locus may also be influenced by a more general genetic drift in these inbred mouse strains [50] and to exclude such a contribution, careful control experiments would be needed. These are becoming technically feasible thanks to advanced molecular biology methods for conditionally induced changes in the genetic set-up at, e.g., the MHC locus. While the behavioural data in mice still would profit from a stronger foundation, the data in humans are still weaker. Thus, the preference for T-shirt odours of HLA-dissimilar donors was not replicated in a larger study [42]. More importantly, there is neither in mice nor men any analytical evidence linking the chemistry of body odours to the genetic set-up at the MHC/HLA locus. Last but not least, no plausible mechanistic explanation was ever presented regarding how the MHC/HLA proteins could affect the secretion of odourant precursors such as the ones summarized in this review: the HLA/MHC specifically bind peptides (at least seven amino acids long, typically nine amino acids) in a peptide-binding groove. Based on the detailed understanding of binding requirements of these proteins, it is rather unlikely that they can bind the odourant precursors described in figure 3 and that different alleles of the HLA would discriminate them and selectively affect the secretion of different odourant precursors to contribute to an individual odourtype. Only if the HLA-bound peptides would themselves act as the odourant principles would a plausible mechanism exist. This has indeed been postulated [51], but its stands in stark contrast to what we know on the physico-chemical nature of odourants in general and on the chemistry of body odours specifically [52]. Thus, even if the hypothesis that odours may communicate information about a ‘matching immune system’ to sire more pathogen-tolerant offspring is tempting, it still needs to answer a number of crucial questions (see also Havlíček *et al*. [53]).

## 11. Odour detection—sensitivity and insensitivity

All the information on the biochemistry presented so far refers to the sender: how odour precursors are produced by the host and how commensal bacteria contribute to finally release the volatiles. Yet, for chemical communication to occur, the receiver is of equal importance. Regarding the receiver, there are two observations of key interest: a particularly high sensitivity to body odourants on the one hand and partial insensitivity on the other hand.

### (a) Sensitivity

Sensitivity to odourants is expressed as the detection threshold measured in ng l^−1^ air— it gives the concentration of an odourant in the vapour phase that a panellist is able to consciously detect. Typical odourants encountered in nature and widely used in perfumery such as the monoterpenes citronellol, geraniol or linalool found in rose or lavender oils have detection thresholds in the range of 2–5 ng l^−1^. For the dominant acid HMHA, we found a threshold of 0.0044 ng l^−1^ [54]—hence, humans have a particularly high sensitivity to this key human-specific odourant, a sensitivity that is higher than the sensitivity to many typical environmental odourants. Similarly, there is a very high sensitivity to the thiols in axilla secretion, and we reported a threshold of 0.001 ng l^−1^ for 3-mercapto-3-methyl-hexanol [7]. This latter sensitivity is less surprising since sulfur compounds, in general, tend to have particularly low detection thresholds. For androstenone, too, a very low detection threshold of 0.0009 ng l^−1^ was reported [55].

In the light of these low detection thresholds, one would postulate specific olfactory receptors with a uniquely high affinity to these particular ligands. Indeed, recently, Noe *et al*. [56] reported OR2M3 as a specific human receptor with a high affinity to 3-mercapto-2-methylpentanol and close homologues. *In vitro* activation of olfactory receptors by odourants is measured in the water phase of cell cultures expressing the receptors and is expressed as EC_50_ values for half-maximal activation in μM—it is currently not fully understood how these values relate to the detection thresholds in the air measured in ng l^−1^. The EC_50_ of 3-mercapto-2-methylpentanol for OR2M3 activation was reported at 0.45 µM, and the EC_50_ values for three investigated homologues correlate to the olfactive detection thresholds. Another receptor, OR7D4, was described as a receptor for androstenone with an EC_50_ of 12 µM [57]. This same receptor is activated with a lower EC_50_ of 2.9 µM by androstadienone. Interestingly, this latter chemical was often used as a reference molecule in human sensory and behavioural studies as a putative human pheromone; however, it is a less pungent odourant as compared to androstenone, especially in females [58]. Thus, the olfactive detection threshold does not appear to correlate to the reported EC_50_ values in this case.

Receptors for the key odourant sweat acids have so far not been reported in the peer-reviewed literature, but a recent patent application describes six odourant receptors responding to 16 investigated straight-chain and branched acids [59]. Interestingly, one receptor (OR52E8) responds among these acids only to HMHA and neither to other investigated acids nor to any of 832 additional odourants screened on this receptor. Thus, it would appear as a highly specific *bona fide* receptor to this human-specific odourant. Surprisingly, the affinity is quite low with a reported EC_50_ of around 100 µM. This presents a puzzle—as either this receptor is much more sensitive in the physiological setting of the olfactory mucosa as compared to the *in vitro* setting or else it is difficult to conceive how breathing air containing 0.01 ng l^−1^ (i.e. a supra-threshold concentration) could rapidly generate a local concentration in the range of the reported EC_50_ in the olfactory mucosa. Thus, it is certainly an area that deserves further research to fully understand how we can detect these body odourants with such a high acuity.

### (b) Insensitivity

The other side of the coin on the sensory side is the reported anosmia for some of the key odourants reported here. For androstenone, a high rate of anosmia was reported in early studies [60]. This high rate has been questioned in more recent studies, and a wide range of different anosmia rates of 1.8–75% were reported [61]. Our institution has reported a lower frequency of 22.4% from in-house studies determined with careful air-dilution olfactometry [55], yet it remains true that a significant proportion of the human population has a low sensitivity to this compound while others are highly sensitive. Similarly, a significant rate of anosmia for 3M2H (21–26%) was also found by air-dilution olfactometry [62]. Also for HMHA, there is a bimodal distribution, with some panellists having high sensitivity with the low detection threshold reported above, and a high anosmia/hyposmia rate of at least 20% was found in parallel. Thus, while humans overall appear particularly sensitive to human odourants, there is also a strikingly high frequency of selective odour blindness to exactly these human odourants!

## 12. In the light of evolution—axilla odour as an evolutionary puzzle

In this review, we tried to give a comprehensive review on what is known of the biochemistry of human axilla odour formation and detection. We have already alluded to the potential evolutionary implications of these findings in the different sections above and will now summarize the key findings in the evolutionary context.

We highlighted the different *specific odourants* that are released as *specific precursors* by *specialized glands*. Combined with the fact that these precursors seem to be produced locally, axillary glands indeed appear to be a specialized metabolic organ producing these compounds for local secretion.

Multiple enzymes and transport proteins of the host are involved in scent release—some of these have already been characterized biochemically with *in vitro* experiments (ABCC11 and GGT1), while others can only be inferred based on what is known on the biosynthesis of related conjugates investigated especially in the toxicological field.

On the receiver side, the high sensitivity of the human nose (and hence the corresponding olfactory receptors) for axillary odourants also indicates a specific adaptation of our olfactory receptor repertoire to these human odourants, although further work is required on this aspect truly to explain the high sensitivity at the receptor level.

Taken together, the above observations on a specific and complex biochemistry for odour formation and detection in the human body suggest that axilla odours must have had an adaptive function in human evolutionary history. We contrasted this observation to other general ‘malodours’ such as foot, faecal or breath odours that can largely be explained by common bacterial catabolism of excess proteins, and no specific evolutionary adaptation is required to lead to such odours as simple by-products of the catabolism of residual proteins by opportunistic bacteria. The fact that our sense of smell is particularly tuned to those odours, too, as warning signals of decay also has evolutionary implications, but that's another story.

Turning from the human host to the commensal bacteria, an interesting case for coevolution could be described. The bacteria have highly specialized enzymes and transport proteins for precursor uptake and odour release. The analysis of the spectrum of odourant precursors released and the substrate specificity of the bacterial enzyme N-AGA reveals a close match of the substrate spectrum offered by the host and the substrate specificity of the bacterial enzyme. This could be further corroborated by the data on the crystal structure of the enzyme described herein for the first time: the Gln residue conserved in all acid precursors is tightly bound by an intricate network of hydrogen bonds in the active site explaining the high substrate specificity for Gln conjugates, while there is ample space for the binding of different hydrophobic residues found in the different substrates offered in this ecological niche by the human host.

We had found that the individual-specific odourtype is stable and genetically determined. This indicates that axilla odours can reveal individual-specific information and could, in principle, contribute to kin recognition. To what extent body odours were indeed a contributing factor in social communication in prehistoric societies may be difficult to assess, yet the stable, genetically determined pattern at least indicates that body odours could have had such a function based on the underlying (bio)chemistry.

While the observations summarized above all support the case for an adaptive function of axillary odours in an evolutionary context, the *ABCC11* mutation, which confers an almost odourless phenotype and that has rapidly spread in human populations in the Far East, is telling an opposing story. The rapid spread of the haplotype containing this mutation points to a strong selection pressure and may indicate that axilla odours had already become an unwanted trait in early agrarian societies living in closer proximity. The reduced sensory capacity to smell the conspecific as revealed by high anosmia rates for body odours points in a similar direction: a loss of importance of chemical communication through axilla odours in recent evolutionary history, which now is also reflected in a widespread use of deodourants in contemporary societies. In Japan, where the frequency of the mutated *ABCC11* haplotype is high, but has not reached 100%, individuals with a functional *ABCC11* allele frequently undergo surgery [63,64] to remove axillary glands. Surgery is even covered by social security as axilla odours owing to a functional *ABCC11* allele are perceived as a disease. Thus, axillary odours appear as a fascinating trace of our evolutionary past having largely lost their role in the contemporary context.

## Data accessibility

The protein structure of N-AGA presented for the first time in this work is deposited at the Protein Data Bank (https://www.rcsb.org/) under deposition number PDB ID 6SLF.
